# Dupilumab: a delayed response in asthmatic and atopic patients treated for chronic rhinosinusitis with nasal polyps

**DOI:** 10.1007/s00405-024-08738-2

**Published:** 2024-06-08

**Authors:** Umberto Tanzini, Andrea Rampi, Alessandro Vinciguerra, Giulia Danè, Mona Rita Yacoub, Mario Bussi, Matteo Trimarchi

**Affiliations:** 1 Division of Head and Neck Department, Otorhinolaryngology Unit, IRCCS San Raffaele Scientific Institute, Milan, Italy; 2 School of Medicine, Vita-Salute San Raffaele University, Milan, Italy; 3 Department of Otolaryngology-Head and Neck Surgery, Ente Ospedaliero Cantonale, Ospedale Regionale di Lugano, Lugano, Switzerland; 4 Otorhinolaryngology and Skull Base Center, AP-HP, Hospital Lariboisière, Paris, France; 5 Unit of Immunology, Rheumatology, Allergy and Rare Diseases, IRCCS Ospedale San Raffaele, 20132 Milan, Italy; 6 Faculty of Biomedical Sciences, Università della Svizzera Italiana, Lugano, Switzerland

**Keywords:** Chronic rhinosinusitis with nasal polyps, Biological therapy, Monoclonal antibody, Asthma, Atopy

## Abstract

**Purpose:**

Chronic Rhinosinusitis with Nasal Polyps (CRSwNP) is a common disease, which was previously approached with sinus surgery or systemic corticosteroids. The advent of biological therapies radically changed the approach to this disease. On the other hand, there is scarce scientific evidence of how specific subsets of patients respond to this treatment.

**Methods:**

this is a monocentric, prospective study investigating the long-term efficacy on biweekly 300 mg dupilumab therapy in CRSwNP, prescribed to 61 patients. Patients were evaluated at baseline and every 2 months for the first 6 months, then at 9, 12, 16, 20 and 24 months.

**Results:**

dupilumab proved to be an effective treatment, neatly improving both subjective and objective measurements in CRSwNP. The main finding of the study is the difference between specific subgroups of patients: while the overall response is similar, patients with Th2 comorbidities such as asthma and atopy tend to reach a stable response later, with the improvement ongoing even after 6 months of therapy, while non-asthmatic, non-atopic patients attain an earlier stability in response.

**Conclusions:**

dupilumab provides an excellent long-term control of CRSwNP, but the response in asthmatic and atopic patients appears to be different and delayed when compared to non asthmatic and non atopic ones.

## Introduction

Chronic rhinosinusitis (CRS) is a common disease, affecting 5–15% of the population. It can be divided into two major phenotypes: chronic rhinosinusitis without nasal polyps (CRSsNP) and with nasal polyps (CRSwNP), with the latter accounting for 25–30% of the cases [1]. CRSwNP treatments include a conservative approach with nasal rinse and intranasal corticosteroids (INCS), but surgery is often required. CRSwNP also tends to have high recurrence rates, and often multiple cycles of systemic corticosteroid or revision ESS are required to provide an improvement in the quality of life, though often just temporary [2]. The introduction of biological therapy with monoclonal antibodies targeting the Type 2 pathway has represented a crucial moment for CRSwNP treatment, providing an effective alternative to revision surgery or corticosteroids [3].

The first monoclonal antibody approved by the Food and Drug Administration (FDA) for CRSwNP was dupilumab, which is a fully human, VelocImmune-derived IgG-4, that blocks the α unit of IL-4 receptor (IL-4R), which is shared with IL-13R, hence inhibiting two key cytokines involved in the persistence of type 2 inflammation. Two major multicentric phase 3 trials (SINUS24 and SINUS52) have shown its excellent and rapid-onset efficacy in improving both subjective and objective measurements of CRSwNP, but only for 52 weeks, while longer follow-ups are still scarce in the literature [4].

Furthermore, now that other biologics such as anti-IgE (omalizumab) and anti-IL-5 (mepolizumab) antibodies are available for CRSwNP [5, 6], it is crucial for clinicians to identify which subset of patients may benefit from the treatment with drug, which one to choose, and, once the therapy is ongoing, when and how to evaluate patient’s response.

These drugs also show their effect in other diseases with type 2 inflammation, such as atopic dermatitis (for dupilumab), and eosinophilic granulomatosis with polyangiitis (mepolizumab), and all three are used in severe asthma [7]. In particular, asthma and CRSwNP are strongly linked, with up to 66% of CRSwNP patients suffering from comorbid asthma, and the presence of both diseases is associated with worse control of either one [8].

This study details our center's experience in treating patients with CRSwNP in the first two years following dupilumab approval in Italy. The main purpose of the study is to confirm the long-term efficacy and safety of dupilumab, but also to show a difference in response timing in specific subgroups of patients, especially asthmatic and atopic patients.

## Materials and methods

This monocentric, observational, prospective trial has been designed to show the efficacy of dupilumab treatment in CRSwNP. This study included all the patients treated in our center with dupilumab 300 mg every two weeks from January 2021 to March 2023, prescribed according to EPOS2020 criteria, for CRSwNP, for a total of 61 patients [1].

All patients were screened with blood tests including cell blood count (CBC), cardiac, kidney and liver function, and an immunological panel; Dupilumab was subsequently prescribed after multidisciplinary discussion with allergologists and immunologists.

The following parameters were evaluated during the baseline visit:

- Assessment of quality of life: (A) Sino-Nasal Outcome Test-22 (SNOT-22) (range 0–110). (B) Visual Analogue Scale for Olfaction (VAS) (range 0–10). (C) The Asthma Control Test (ACT) was obtained in asthmatic patients (range 5–25).
- Endoscopic evaluation: (a) NPS (Nasal Polyp Score) (range 0–4 for each nasal fossa) (b) Lund-Kennedy score (LKS) (range 0–10 for each nasal fossa). LKS was employed as it also considers parameters such as secretion, scarring and edema, and therefore provides a more general view of the grade of inflammation when compared to NPS.
- Radiological results on the most recent CT scan: (A) Lund-Mackay score (LMS). (B) ACCESS rating. Both have a scale of 0 to 24 [9].
- Previous medical history: (A) Comorbidities, with particular emphasis on asthma and atopic dermatitis (AD). (B) Acetylsalicylic acid (ASA) or other nonsteroidal anti-inflammatory medication sensitivities or intolerance (NSAIDs). D) The number of EPOS 2020 minor criteria that have been met (ranging from 3 to 5). (C) number and timing of previous surgeries.
- Recent measurements of Fractional Exhaled Nitric Oxide (FENO) were registered, when possible.

The follow-up included a visit every two months for six months, then at 9, 12, 16, 20 and 24 months. During these visits, the ENT surgeon reevaluated the aforementioned parameters during endoscopic examination, and the questionnaires on the quality of life were resubmitted. Blood tests (including CBC, total IgE and CPR) were repeated for each visit, especially to routinely monitor blood eosinophilia [10, 11]. At 4–6 and 12 months, EPOS2020 response criteria were reassessed.

### Statistical analysis

Statistical analysis was performed with SPSS version 26 (IBM Corp. in Armonk, NY, USA). Data are reported as mean ± standard deviation or percentage, as appropriate. Statistical significance is considered for p < 0.05. Statistical analysis was performed using paired sample analysis (T-Student test), by confronting, for all parameters (NPS, SNOT-22, LKS, olfaction VAS), the difference between the value at baseline and at 2 months, then the difference between the value at 2 months and 4 months, and so on: we decided to perform our analysis because the long-term improvement compared to the baseline was surely statistically significant once improved at 2 months.

## Results

This study included 61 (39M, 22 F) patients treated with Dupilumab and at least 6-month follow-up, of which 42 patients reached at least 9 months, 27 reached 12 months, and 9 reached 24 months. The median age was 52 years (range 26–84). 47 patients (77%) suffered from asthma, 26 (42%) had aspirin or NSAID intolerance and 32 were atopic (52%). All patients previously underwent FESS, with a median number of 2 surgical operations (range 1–15). Mean pre-therapy Lund-Mackay and ACCESS scores were respectively 19 (range 7–24) and 8 (range 1–20). Pre-therapy FENO level was available in 20 patients, with a mean level of 81,6 ppb. All values are reported in Table 1.

**Table 1:** Mean valutes of the variables found at baseline

Variables (n = 61)	Values
Age	52.8 (26–84)
Sex	39 M, 22 F
Asthma	47 (77%)
Atopy	32 (52%)
N-ERD	26 (42%)
Eosinophil absolute count (EAC)	540 (100–1200)
Total IgE	158 (3.9–1726)
Lund-Mackay Score	20.0 (7–24)
ACCESS score	6.4 (0–20)
Number of previous surgeries	2.2 (1–15)
FENO level	81.6 (7–270)

In brackets, the minimum and maximum value. Eosinophil absolute count is expressed in cells/μl; total IgE in UI/L; FENO in parts per billion (ppb). For sex, the number of male (M) and female (F) patients is reported. For asthma, atopy and N-ERD, we reported the total of patients and the percentage in brackets for that specific comorbity

N-ERD: NSAIDs-exacerbated respiratory disease

All subjective and objective measurements showed a progressive improvement during treatment, as reported in Table 2. All subjective and objective measurements showed a progressive improvement during treatment: Pre-treatment mean SNOT-22 was 62.8, while at 2–4–6–9–12 and 20 months was respectively 25.2–18.4–15.3–12.5–10.2 and 8.3. Similarly, olfaction VAS pre-treatment and at 2–4–6–9–12 and 20 months was 0.2–4.9–5.9–6.8–6.8–7.6 and 8.2 NPS and Lund-Kennedy pre-treatment and at 2–4–6–9–12 and 20 months were respectively 5.7–3.2–2.3–1.9–1.5–1.0 and 0.82 and 10.3–6.6–5.3–4.2–3.4–3.0 and 2.6. We found that all four parameters (SNOT-22, VAS olfaction, NPS, Lund-Kennedy) have a significant improvement for the first six months (p < 0.05), and then the response tends to stabilize.

**Table 2:** Median value of the main variables at baseline and at 2–4–6–9–12–16–20 months Data are reported as median value, [standard deviation] and (p-value)

	Baseline (n = 60)	2 months (n = 58)	4 months (n = 56)	6 months (n = 54)	9 months (n = 42)	12 months (n = 27)	20 months (n = 19)
SNOT-22	62.6, [17.0]	**25.2, [15.1] (< 0.001)**	**18.4, [14.5]** **(< 0.001)**	**15.3, [13.2]** **(0.011)**	12.5, [9.9] (0.07)	10.2,[7.7] (0.09)	8.3, [9.4] (0.14)
NPS	5.7, [1.6]	**3.2, [1.6]** **(< 0.001)**	**2.3, [1.7] (< 0.001)**	**1.9, [1.7]** **(0.02)**	**1.4, [1.5]** **(0.04)**	1.0,[1.3] (0.14)	**0.8 [1.4]** **(0.02)**
LKS	10.3, [2.6]	**6.6, [2.2] (< 0.001)**	**5.5, [2.5] (0.001)**	**4.2, [2.5] (0.01)**	**3.4, [2.3] (0.03)**	3.0, [2.4] (0.38)	2.9, [2.4] (0.65)
Olfaction VAS	0.2, [0.9]	**4.9, [3.2] (< 0.001)**	**5.9, [3.2] (< 0.001)**	**6.9, [3.3] (< 0.001)**	6.9, [3.3] (0.06)	7.3, [3.0] (0.11)	8.2, [2.5] (0.07)

SNOT-22: SinoNasal Outcome Test-22; NPS: Nasal Polyp Score; LKS: Lund-Kennedy Score; VAS: Visual Analog Scale

The p-value shown refers to the significavity of difference with the previous value. Bold values denote statistical significance at the p < 0.05 level

**Fig. 1:**
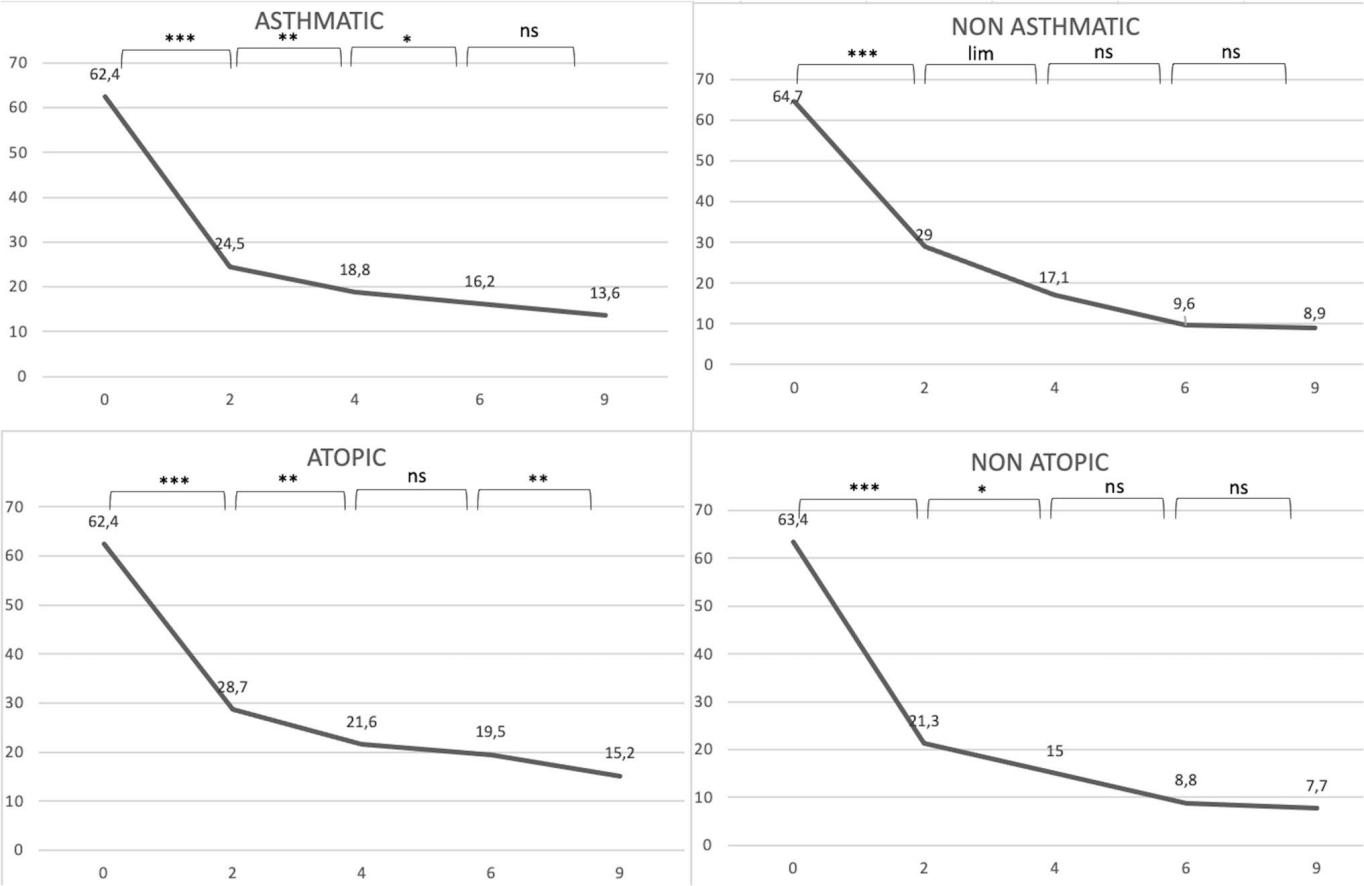
Median values of SNOT-22 at 0, 2, 4, 6 and 9 months. ***denotes a p < 0.001, **a p < 0.01, *a p < 0.05, lim a p-value of 0.05–0.06, ns a p > 0.06

**Fig. 2:**
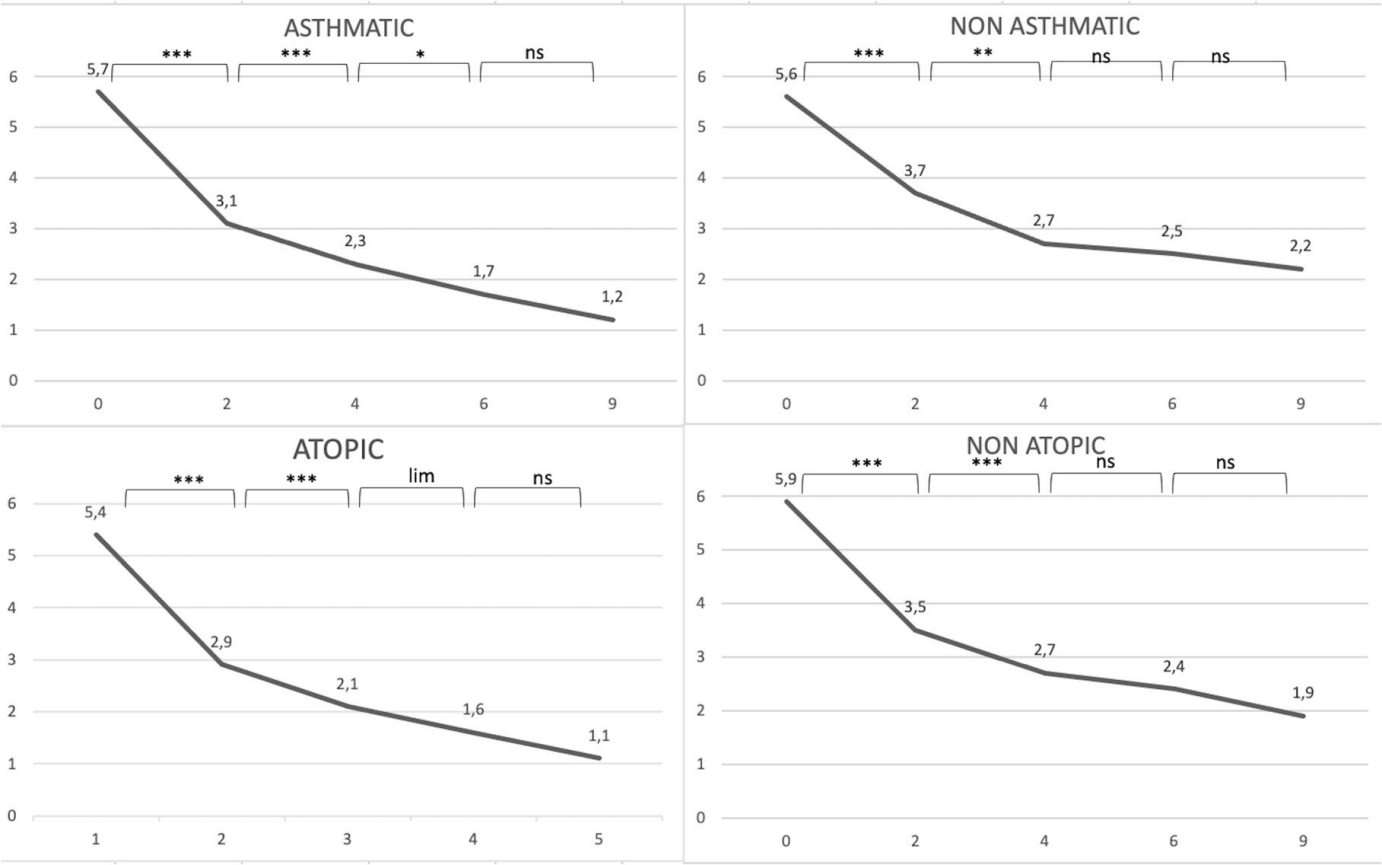
Median values of NPS at 0, 2, 4, 6 and 9 months. ***denotes a p < 0.001, **a p < 0.01, *a p < 0.05, lim a p-value of 0.05–0.06, ns a p > 0.06

**Fig. 3:**
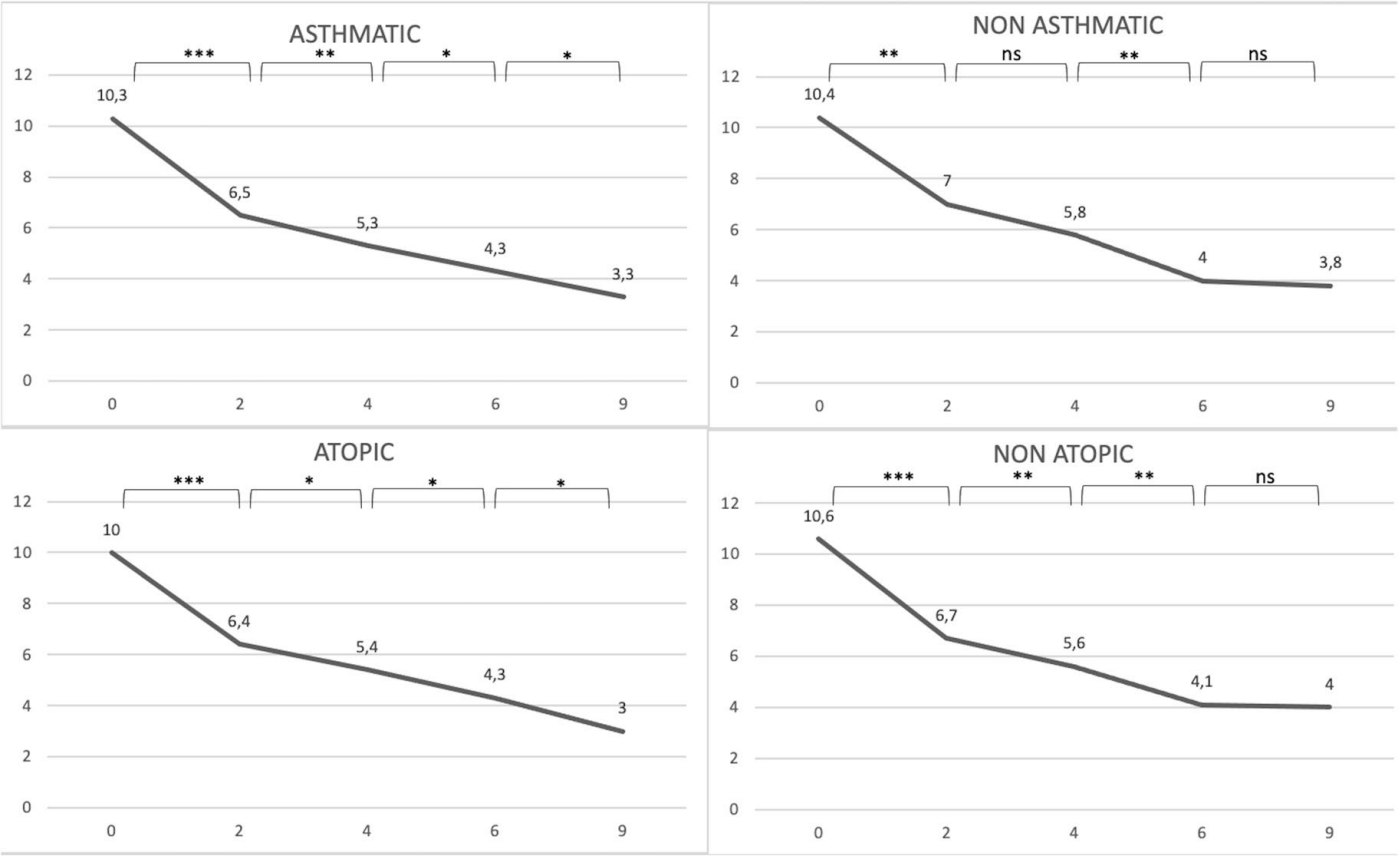
Median values of Lund-Kennedy score at 0, 2, 4, 6 and 9 months. ***denotes a p < 0.001, **a p < 0.01, *a p < 0.05, lim a p-value of 0.05–0.06, ns a p > 0.06

**Fig. 4:**
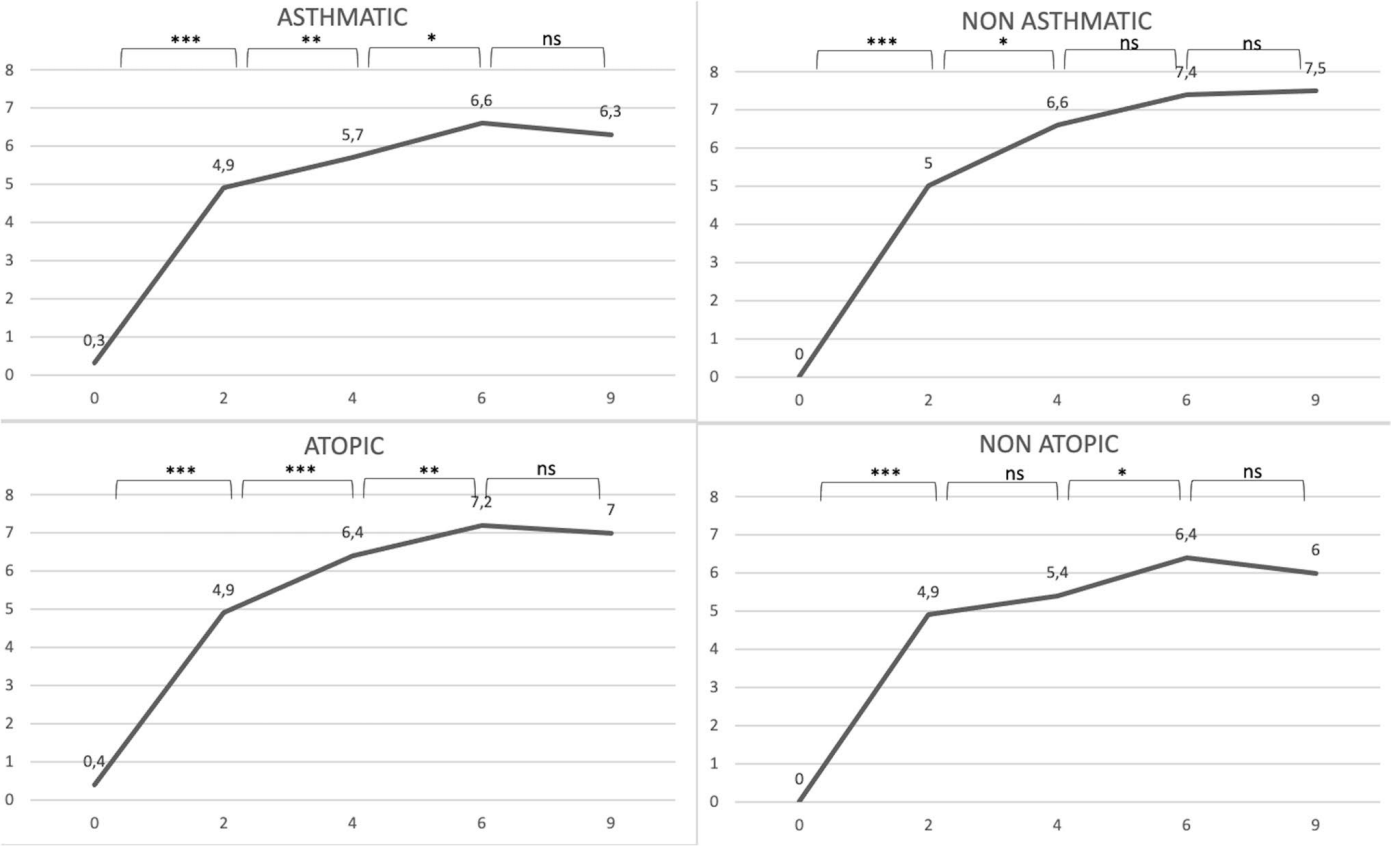
Median values of olfaction VAS at 0, 2, 4, 6 and 9 months. ***denotes a p < 0.001, **a p < 0.01, *a p < 0.05, lim a p-value of 0.05–0.06, ns a p > 0.06

The same analysis was then performed, but splitting asthmatic patients from non-asthmatic ones. No difference between the groups of asthmatic and non astmatic patients at baseline for the four considered parameters, as well as between the groups of atopic and non atopic ones. We subsequently analyzed the trend of response within each of the subgroups, in order to determine the timing at which patients stopped improving significantly, in other words achieving a steady response to dupilumab. We found that non-asthmatic patients tend to present a stability in response more precociously than astmatic ones: the results tend to reach a plateau around 4 months after the beginning of treatment, then the difference between values become non-significant (SNOT-22, VAS olfaction, NPS p-values for the difference between 6 and 4 months were respectively 0.11, 0.12, 0.34. Lund-Kennedy still improved, with a p-value of 0.005) while asthmatic patients reach that plateau later, with all parameters improving significantly at 6 months and some even at 9 months (Lund-Kennedy p-values for the difference between 9 and 6 months were respectively 0.07, 0.08 and 0.01, while SNOT-22 and NPS have p-values at the limit of statistical significance, respectively 0.07 and 0.08). A similar pattern of more tardive stabilization in response was found in atopic patients, with non-atopic patients showing a progressive improvement that doesn’t increase significantly at six months (SNOT-22, VAS olfaction, NPS and Lund-Kennedy p-values for the difference between 9 and 6 months were respectively 0.840, 0.176, 0.134 and 0.674), while atopic patients still showed significant response in some parameters at 9 months (SNOT-22 and Lund-Kennedy p-values for the difference between 9 and 6 months were respectively 0.007 and 0.02). All data are reported as graphs in Figs. 1, 2, 3 and 4.

## Discussion

The introduction of biologics in CRSwNP has marked an actual turning point in the treatment of the disease. Dupilumab was the first one approved by the FDA for CRSwNP, and it has shown its efficacy in improving the vast majority of subjective and objective findings, both in clinical trials and real-life settings [4, 12].

This study confirms a significant effect on all parameters commonly used during CRSwNP follow up: patient reported outcomes such as SNOT-22 and olfaction VAS and endoscopic parameters such as NPS and LKS showed a remarkable improvement, as reported in Table 2. Results tend to be achieved rapidly, in fact all parameters get better after just 2 months. Most tend to reach stability after 6 months, even though some (especially NPS) show continuous improvement up to 20 months after the beginning of the treatment. Some studies remark even faster improvement, after only one dose of dupilumab [13].

The main finding of this study, that hasn’t been observed before, is a difference in the kinetic of response to dupilumab in various subgroups of patients. We observed that patients with more prominent Th2 comorbidities, that is asthma and atopy, tend to have a more prolonged response in all measurements, both subjective and objective. While the overall response is similar in these subgroups, in fact, the response in non-asthmatic, non-atopic patients tend to have a more precocious response and stabilize more rapidly, around 4–6 months, while asthmatic or atopic patients have a significant response at 6 and even 9 months after the beginning of the treatment. It is well established that CRSwNP with comorbid asthma and atopy is often more severe and associated with an increased probability of postoperative recurrence [8]. Higher levels of specific markers of eosinophilic inflammations, such as total IgE and FENO, also represent a risk factor for relapsing form of nasal polyposis [14]. IL-4, whose receptor is directly inhibited by dupilumab itself, plays a key role in Th2 differentiation of lymphocytes and IgE production, and it has been shown to be further increased in atopic patients with nasal polyposis compared to non atopic ones [15]. A study by Nabavi et al. showed that IL-13, the other target of dupilumab inhibition, was higher in CRSwNP patients than healthy controls. Furthermore, IL-13 was significantly increased in patients with comorbid asthma [16]. IL-4 and IL-13 also directly induce the production of FENO [17], which is commonly used as a predictive factor for exacerbation recurrence in asthma [18], but it isn’t considered in most CRSWNP guidelines [19]. That could explain the reason of the prolonged response: in patients with high Th2 inflammatory load, a complete clinical regression to dupilumab might be delayed, but still achieving an optimal response. A recent multicentric study by De Corso et al. [20] that included part of our data, has similarly analyzed the difference in response in asthmatic and non-asthmatic patients. The authors point out a faster reduction in SNOT-22 and NPS for asthmatic patients, which isn’t found in our cohort of patients. On the other hand, while not explicitly stated in the paper, it’s possible to observe that asthmatic patients have a significant response between 6 and 3 months in both SNOT-22 and NPS, while non-asthmatic patients don’t. That finding confirms our hypothesis, even when investigating a larger cohort, that includes more than 600 patients.

Other studies have tried to identify specific subgroups that might benefit from a better or faster response to dupilumab. Guo et al. [21] analyzed biomarkers that might indicate a better response to dupilumab, especially serum osteoprotegerin levels at baseline. Bertlich et al. [22] compared response in patients according to the presence of AERD, histologic eosinophilia or increased blood eosinophil or IgE-levels, without finding difference in the subgroups. In our study, we didn’t focus on a single biomarker, but rather on the global burden of disease in patients with type 2 inflammation with different related pathologies, which often present multiple increased values simultaneously (eg. AERD, blood eosinophilia and increased IgE level are more common in asthmatic patient). Furthermore, dupilumab response is quite fast, so the effect was evident when considering the first months of treatment, while the aforementioned study [22] only repeated follow up every three months.

This aspect is fundamental to consider when assessing the response: most guidelines suggest an evaluation at 6 and 12 months after the beginning of the therapy, [19, 23, 24] but some others suggest evaluation as early as 4 months (such as EPOS2020 guidelines [1]), and no guidelines suggest a different timing in specific subgroups of patients, potentially underestimating the benefit of patients with a delayed complete response to biologics.

Another fundamental point is the difficulty in selecting the patients that should begin this kind of treatment.

Our study, in accordance with the other trials available in literature [4, 12, 20, 25], once again confirms the effectiveness of dupilumab, with a substantial improvement in the quality of life (SNOT-22 questionnaire), nasal polyps’ dimension, overall nasal mucosal inflammation (measured with NPS and Lund-Kennedy score) and olfactory function. However, there are some points that demand attention, mainly the presence of non-responder patients and the difference in the kinetic of response.

Out of the 61 patients, one discontinued the treatment due to an almost complete lack of response: after one year of treatment, NPS didn’t change from baseline and SNOT-22 improved by only 12 points. It is important to notice that this patient was the only one in our cohort that didn’t show a serum evidence of Th2 inflammation and didn’t suffer from comorbid asthma, a typical Th2 comorbidity. In most guidelines, especially in European ones, [1, 19, 23, 24, 26] the evidence of Th2 inflammation is an optional criterion, but it is important to point out that patient without that kind of evidence might not benefit from biological treatment.

Another hot topic in biologics treatment is the decision to proceed with their prescription or with revision surgery. While some guidelines contemplate the possibility of prescription even in patients without previous surgery [26], we decided to institute a treatment with dupilumab only in previously operated patients. Full-house FESS allows to increase the portion of mucosa reachable by intranasal corticosteroids (INCS), that are proven to consistently improve patients’ symptoms and post-operative recurrence of nasal polyps, [27] hence potentially providing long-term control of the disease without the need for biological therapy. On the other hand, the extent of previous surgery is not well established. Some authors, especially in countries where the access to biological therapies is limited, tend to prefer revision surgery with an extended approach (such as Draf III procedure for frontal sinus or reboot surgery [28]). In this study, due to the heterogeneity of data, we could not prove statistically that the extent of surgery (measured through the ACCESS score [29]) was indifferent for the response, but we didn’t observe a qualitative difference in dupilumab response when comparing high scores of ACCESS to low ones. This study, however, presents some limitations: the prospective nature of the study, while granting an homogeneous analysis of each patient, leads to missing data that couldn’t be recovered retroactively. This is mainly important considering atopic patients: we didn’t routinely screen patients for allergen sensitization at baseline and, while most underwent an evaluation with an allergologist in our centre, some patients were followed by a pulmonologist for asthma, hence possibly underestimating the prevalence of atopy. Furthermore, while having access to more than 60 patients’ data, more than 75% of them were asthmatic, so the comparison between asthmatic and non-asthmatic patients might be skewed due to the intrinsic difference in the numerosity of the subgroups.

## Conclusions

Our findings support the efficacy and safety of dupilumab even during a longer follow-up for all the main aspects of CRSwNP, consistently with the other studies in the literature and with the findings of other diseases treated with dupilumab. Furthermore, this study suggests that there might be differences in asthmatic and atopic patients, requiring a longer follow-up to best evaluate response to treatment.

## Data Availability

The data that support the findings of this study are available from the corresponding author, MT, upon reasonable request.
